# National Association of Dental Examiners

**Published:** 1891-10

**Authors:** 


### National Association of Dental Examiners.

The National Association of Dental Examiners held its tenth annual session at Saratoga Springs, commencing Monday, August 3, 1891.

In the absence of the regularly elected officers, Dr. L. D. Shepard was elected president and Dr. Levy secretary-treasurer pro tem.

The following State Boards of Dental Examiners were represented:

Vermont. James Lewis.

New Jersey. Fred. C. Barlow, Fred. A. Levy.

Ohio. J. Taft, H. A. Smith, L. E. Custer, C. R. Butler.

Pennsylvania. Louis Jack, W. E. Magill.

Georgia. George W. McElhaney.

Maryland. A. J. Volck.

Massachusetts. L. D. Shepard, J. S. Hurlbut.

Mississippi. W. H. Marshall.

Iowa. J. T. Abbott.

Louisiana. Geo. J. Friedrichs.

The following State Boards were admitted to membership:

Tennessee. J. Y. Crawford.

New Hampshire. E. B. Davis.

Main. E. J. Roberts, D. W. Fellows.

A committee consisting of Drs. Shepard, Fellows, Crawford, McElhaney and Magill was appointed to confer with a similar committee from the National Association of Dental Faculties for the better understanding of questions involving educational interests.

The committee subsequently reported that the conference committees had agreed upon the following resolutions, which were, on motion, confirmed:

WHEREAS, There can be no question that the main object in view of both the National Association of Dental Faculties and the National Association of Dental Examiners is the better preparation of young dentists for usefulness in the community, and that to secure this end it is desirable that the State boards of dental examiners and the colleges should work in harmony; therefore,

*Resolved*, That it is recommend to the State boards that when a graduate after examination has been refused a license and his college request information as to the causes of his failure to pass the examination, the boards shall furnish the faculty with a detailed statement of the subjects and questions on which the applicant has failed.

*Resolved*, That we discountenance the publication by the State boards of the names of colleges whose graduates have failed to pass.

The committee on colleges reported that they had received reports of the number of matriculates and graduates of twenty-eight colleges, as shown below:

### Number of Matriculates and Graduates of the Dental Colleges.

| College | Location | Matriculates. | Absentees. | Graduates. | Ratio. |
|---|---|---|---|---|---|
| Baltimore College of Dental Surgery | Baltimore, Md. | 224 | 3 | 76 | 34.3 |
| Boston Dental College | Boston, Mass. | 96 | | 31 | 32 2 |
| Chicago College of Dental Surgery | Chicago, Ill. | 323 | | 94 | 29. |
| Harvard University, Dental Department | Boston, Mass. | 44 | | 15 | 34. |
| Kansas City Dental College | Kansas City, Mo. | 110 | 5 | 43 | 40. |
| Missouri Dental College | St. Louis, Mo. | 90 | | 26 | 28.8 |
| New York College of Dentistry | New York, N. Y. | 283 | 8 | 85 | 30.9 |
| Ohio College of Dental Surgery | Cincinnati, Ohio. | 208 | | 75 | 36. |
| *Pennsylvania College of Dental Surgery | Philadelphia, Pa. | 252 | 17 | 94 | 40. |
| †Philadelphia Dental College | Philadelphia, Pa. | 315 | | 146 | 46.3 |
| University of California, Dental Department | San Francisco, Cal. | 63 | | 16 | 25.4 |
| University of Iowa, Dental Department | Iowa City, Ia. | 161 | | 58 | 36. |
| University of Michigan, Dental Department | Ann Arbor, Mich. | 132 | 1 | 29 | 22.1 |
| University of Pennsylvania, Dental Department | Philadelphia, Pa. | 206 | 3 | 83 | 40.8 |
| Vanderbilt University, Dental Department | Nashville, Tenn. | 135 | | 43 | 31 8 |
| Northwestern College of Dental Surgery | Chicago, Ill. | 14 | | 3 | 21.4 |
| Indiana Dental College | Indianapolis, Ind. | 96 | | 40 | 41.6 |
| Dental Department of Southern Medical College | Atlanta, Ga. | 103 | | 38 | 36.8 |
| School of Dentistry of Meharry Medical College, Department of Central Tennessee College | Nashville, Tenn. | 5 | | 1 | 20. |
| University of Maryland, Dental Department | Baltimore, Md. | 163 | 4 | 64 | 39.7 |
| Columbian University, Dental Department | Washington, D. C. | 19 | | 2 | 10.5 |
| Royal College of Dental Surgeons of Ontario | Canada. | 67 | | 27 | 40. |
| College of Dentistry, Department of Medicine, University of Minnesota | Minneapolis, Minn. | 36 | | 7 | 19.4 |
| American College of Dental Surgery | Chicago, Ill. | 167 | | 49 | 29. |
| Aggregate | | 3312 | | 1144 | 34.5 |

*15 female matriculates. †9 female matriculates.

### Dental Colleges not Connected with the National Association.

| College | Location | Matriculates. | Absentees. | Graduates. | Ratio. |
|---|---|---|---|---|---|
| German-American | Chicago, Ill. | 22 | | 11 | 50. |
| Western Dental College | Kansas City, Mo. | 61 | 1 | 9 | 15. |
| United States Dental College | Chicago, Ill. | 43 | | 11 | 25 5 |
| College of Dentistry, University of Denver | Denver, Col. | 12 | | 5 | 41.6 |
| Aggregate | | 139 | | 36 | |
| Whole aggregate | | 3451 | | 1180 | |

Four colleges, members of the National Association of Dental Faculties, had failed to report, namely: University Dental College, Chicago; Dental Department, University of Tennessee; Louisville College of Dentistry; and Dental Department of National University, Washington, D. C.

The committee recommended that " as the next session of the colleges marks the commencement of the new plan of three years' college instruction, that this association request the National Association of Dental Faculties to require each school to issue each year an announcement containing a list of the students, classified in the three grades of seniors, juniors and freshmen; that this list also in each instance designate the absentees, and that each school be required in the same announcement to publish a list of the graduates of the preceding session."

The committee also reported the following list of colleges which they recommend as reputable:

Baltimore College of Dental Surgery, Baltimore, Md.

Boston Dental College, Boston, Mass.

Chicago College of Dental Surgery, Chicago, Ill.

College of Dentistry, Department of Medicine, University of Minnesota, Minneapolis, Minn.

Dental Department, Columbian University, Washington, D. C.

Dental Department of Northwestern University (now the University Dental College) Chicago, Ill.

Dental Department of Southern Medical College, Atlanta, Ga.

Dental Department of University of Tennessee, Nashville, Tenn.

Harvard University, Dental Department, Cambridge, Mass.

Indiana Dental College, Indianapolis, Ind.

Kansas City Dental College, Kansas City, Mo.

Louisville College of Dentistry, Louisville, Ky.

Missouri Dental College, St. Louis, Mo.

New York College of Dentistry, New York City.

Ohio College of Dental Surgery, Cincinnati, Ohio.

Pennsylvania College of Dental Surgery, Philadelphia, Pa.

Philadelphia Dental College, Philadelphia, Pa.

School of Dentistry of Meharry Medical Department of Central Tennessee College, Nashville, Tenn.

University of California, Dental Department, San Francisco, Cal.

*Northwestern College of Dental Surgery, Chicago (defunct).

University of Iowa, Dental Department, Iowa City, Ia.

University of Maryland, Dental Department, Baltimore, Md.

University of Michigan, Dental Department, Ann Arbor, Mich.

University of Pennsylvania, Dental Department, Philadelphia, Pa.

Vanderbilt University, Dental Department, Nashville Tenn.

Western Dental College, Kansas City, Mo.

Minnesota Hospital College, Dental Department, Minneapolis, Minn. (defunct).

St. Paul Medical College, Dental Department, St. Paul, Minn. (defunct).

The report was adopted, and the committee on schools was enlarged to consist of one member from each State board. Dr. Louis Jack was appointed chairman of the committee, and the president, secretary and chairmen were authorized to complete its membership and to fill vacancies.

The following was adopted:

*Resolved,* That this association requests the several boards represented in this body not to indorse beneficiary students.

The following officers were elected for the ensuing year: L. D. Shepard, President; W. E. Magill, Vice-President; Fred. A. Levy, Secretary-Treasurer.

The officers were empowered to select the time and place of the next meeting.

Adjourned.

